# Why dispersal should be maximized at intermediate scales of heterogeneity

**DOI:** 10.1007/s12080-012-0171-3

**Published:** 2012-09-27

**Authors:** Peter Skelsey, Kimberly A. With, Karen A. Garrett

**Affiliations:** 1 Department of Plant Pathology, Kansas State University, Manhattan, KS 66506 USA; 2 Division of Biology, Kansas State University, Manhattan, KS 66506 USA

**Keywords:** Movement, Spatial heterogeneity, Population connectivity, Patchiness, Habitat fragmentation, Matrix resistance

## Abstract

Dispersal is a fundamental biological process that results in the redistribution of organisms due to the interplay between the mode of dispersal, the range of scales over which movement occurs, and the scale of spatial heterogeneity, in which patchiness may occur across a broad range of scales. Despite the diversity of dispersal mechanisms and dispersal length scales in nature, we posit that a fundamental scaling relationship should exist between dispersal and spatial heterogeneity. We present both a conceptual model and mathematical formalization of this expected relationship between the scale of dispersal and the scale of patchiness, which predicts that the magnitude of dispersal (number of individuals) among patches should be maximized when the scale of spatial heterogeneity (defined in terms of patch size and isolation) is neither too fine nor too coarse relative to the gap-crossing abilities of a species. We call this the “dispersal scaling hypothesis” (DSH). We demonstrate congruence in the functional form of this relationship under fundamentally different dispersal assumptions, using well-documented isotropic dispersal kernels and empirically derived dispersal parameters from diverse species, in order to explore the generality of this finding. The DSH generates testable hypotheses as to when and under what landscape scenarios dispersal is most likely to be successful. This provides insights into what management scenarios might be necessary to either restore landscape connectivity, as in certain conservation applications, or disrupt connectivity, as when attempting to manage landscapes to impede the spread of an invasive species, pest, or pathogen.

## Introduction

Dispersal is essential for maintaining population connectivity, which in turn is important for mitigating extinction risk and the loss of genetic diversity within populations (With and King 1999; Ezard and Travis 2006). For many species, population connectivity has already been seriously disrupted through the wholesale loss and fragmentation of their habitat as a result of human land-use activities (Millennium Ecosystem Assessment 2005a, c). The restoration of population connectivity, such as by the creation of dispersal corridors among isolated habitat fragments, is thus frequently advocated in conservation planning and reserve design (Crooks and Sanjayan 2006). Conversely, some of the most important environmental and public health threats we now face are ultimately a consequence of “over-connectivity,” in which the spread of a non-native species or disease has been facilitated by human activity, leading to increased homogenization of biotas and an elevated risk of global pandemics (Millennium Ecosystem Assessment 2005b). To address the threats of invasive or disease spread, control measures might seek to disrupt dispersal—and thus connectivity among sites or hosts—through landscape management techniques, involving targeted habitat removal or an increase in spatial heterogeneity (e.g., using mixed cultivars or inter-cropping in agricultural fields) to slow the rate of invasive spread by effectively increasing the distance among susceptible host populations (Glass et al. 2002; Gomez et al. 2008; Mills et al. 2010; Skelsey et al. 2010; Swei et al. 2011).

Clearly, dispersal can have different implications for landscape management depending on the mode of spread or transport, the nature of the landscape through which the organism moves, and whether its spread is deemed beneficial, neutral, or harmful to humans or other species. Typically, however, the different sorts of interactions between dispersal and spatial heterogeneity have been explored independently in different disciplines, which reinforce the perception that there must be something fundamentally different about how, for example, a bird versus a plant pathogen disperses across the landscape. If we ignore the obvious differences in terms of their specific mode of dispersal or the distance length scales over which it occurs, however, might it nevertheless be possible to abstract a general scaling relationship for these different systems that would enable us to predict under what landscape scenarios dispersal is maximized? Such scaling laws are ubiquitous in other areas of ecology (e.g., Brown et al. 2002), and would facilitate identification of the particular scales at which spatial heterogeneity (landscape structure) should be targeted for the management and the regulation of population connectivity.

In considering the relationship between spatial heterogeneity and dispersal, we are confronted with a fundamental question: “At what spatial scale(s) is dispersal among habitat patches maximized?” This is an important question as identifying the scales at which dispersal is maximized (and by corollary, reduced) may be a crucial factor in determining how to manage landscapes for enhanced or reduced dispersal, as when restoring habitat connectivity to facilitate dispersal among isolated populations or conservation reserves (where connectivity is generally beneficial) or when disrupting connectivity to prevent the spread of disease or an invasive species (where connectivity is harmful). Whilst dispersal among patches tends to increase with an increase in the amount of habitat (or hosts, as the case may be) and with the degree of habitat (or host) aggregation (With and King 1999; King and With 2002), these results regarding the effect of habitat on dispersal are in fact scale dependent. The effects on dispersal are constrained by the scale of the landscape, both in terms of the spatial grain or fine-scale resolution of the landscape pattern (patch size) as well as the distance separating habitat patches (patch isolation). Assuming that population density is proportional to habitat area, this can lead to conflicting hypotheses as to when and at what scales dispersal—and thus connectivity—is maximized. If we scale the entire system relative to the organism, then at finer spatial scales, the distance separating habitat patches tend to be smaller, which should facilitate dispersal among those patches. Conversely, at broader spatial scales, the distance between habitat patches tend to be larger, which decreases the likelihood of dispersal among patches beyond some critical distance. From this, we might conclude that dispersal among patches should be maximized at fine spatial scales. However, smaller habitat patches contain and produce relatively fewer dispersal agents (e.g., populations are smaller in small patches), which may reduce dispersal in terms of numbers of individuals or propagules that disperse. In contrast, larger habitat patches contain or can produce relatively more dispersal agents, and also provide larger “targets” that can accumulate or attract more dispersers (e.g., larger habitat patches are perceived to have more resources), which increase the likelihood that agents will arrive at these more distant patches. This then suggests that dispersal among patches should instead be maximized at broader spatial scales. Thus, we have contrasting hypotheses as to the scale of spatial heterogeneity at which dispersal among patches is most likely to be maximized. To complicate matters further, there may not be a positive correlation between patch size and the distance between patches at different scales; larger habitat patches can be separated by smaller distances, and smaller patches by greater distances.

The idea of scale-dependent relationships in movement responses to landscape structure is not new. For example, under the gradient paradigm of landscape structure (Whittaker 1967), successful movement is influenced by environmental gradients that vary with distance from a source (Janzen 1970; Connell 1971). Further, functionally based definitions of landscape connectivity emphasize that a given landscape will be differentially permeable to organisms (i.e., connected) depending upon the scale of dispersal relative to the scale of heterogeneity (Taylor et al. 1993; With et al. 1997; Brooks 2003; Baguette and Van Dyck 2007). In network-based approaches, connections between network elements (individuals, habitat patches, or populations) can be examined at different spatial scales to evaluate the effect on overall network connectivity (Keitt et al. 1997; Bunn et al. 2000; Brooks et al. 2008; Margosian et al. 2009). Thus, while it is recognized that there may exist optimal scales at which to investigate or manipulate ecological flows (Betts et al. 2006; Betts et al. 2007; Pinto and Keitt 2008; de Knegt et al. 2011), to our knowledge, a comprehensive model of how the expected relationship between spatial heterogeneity and dispersal changes across a range of scales has not yet been developed.

**Fig. 1:**
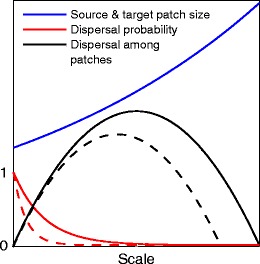
Conceptual representation of the dispersal scaling hypothesis (DSH). Scaling a heterogeneous (patchy) environment relative to the gap-crossing abilities of a species produces antagonistic “dispersal forces” that result in scale-dependent shifts in the magnitude of dispersal (number of individuals) among patches

Here, we present a new framework—the dispersal scaling hypothesis (DSH)—that posits that the magnitude of dispersal (number of individuals) among patches (hereafter, dispersal) should be maximized when the scale of spatial heterogeneity (defined in terms of patch size and isolation) is neither too fine nor too coarse relative to the gap-crossing abilities of an organism (i.e., the ability or willingness to traverse gaps — the intervening matrix between patches). Conceptually, we can envision this emerging as a consequence of antagonistic forces operating at different scales (Fig. 1). As the scale of landscape pattern increases relative to the organism (i.e., a coarse-grain patch structure), the size of habitat patches and the quantity of dispersal agents both increase (larger patches = larger populations = more dispersers). Similarly, larger habitat patches also make bigger “targets,” or are perceived to be more attractive to dispersers. This produces a “positive dispersal force” that increases dispersal over longer distances (Fig. 1; blue line). However, the distance between patches (patch isolation) will ultimately increase, eventually exceeding the gap-crossing abilities of the species. This decreases the probability of movement among patches, resulting in an accompanying “negative dispersal force” (Fig. 1; red line). Rescaling of landscape pattern thus changes these competing dispersal forces, but to different degrees, resulting in scale-dependent shifts in dispersal. Dispersal is predicted to be maximized when the product of these opposing forces is at its maximum positive value: number of redistributed agents, *y* (number) = source strength (number) × dispersal probability (number per square meter) × receptor area (square meter). This maximum will occur when the scale of spatial heterogeneity is neither too fine nor too coarse relative to the gap-crossing abilities of the organism; that is, at an intermediate range of scales (Fig. 1).

The DSH further predicts that intermediate-scale optima in dispersal will still exist for varying degrees of patch isolation. For example, halving or doubling the degree of patch isolation will tend to have a limited effect on dispersal when the pattern of habitat patches is fine-grained relative to the gap-crossing abilities of the organism, as gaps are easily traversed regardless. By corollary, when the pattern of habitat patches is coarse-grained relative to the organism, gaps are less easily traversed and halving or doubling the degree of patch isolation should have a greater effect on dispersal. Thus, a parabolic relationship between dispersal and scale of heterogeneity should still exist, although the interplay between the gap-crossing abilities of the organism and degree of patch isolation will affect the scale at which optimum dispersal occurs (Fig. 1; solid versus dashed lines).

In this paper, we develop an analytical approach that incorporates scaling of spatial heterogeneity (both in terms of patch size and patch isolation) to predict the spatial scale at which dispersal is maximized. We parameterized this model for a range of ecologically important species representing vastly different modes of dispersal, using empirically derived dispersal kernels from the literature, so as to ground our results in ecological reality. We show that, regardless of the species or dispersal process in question, dispersal is maximized at an intermediate range of scales of spatial heterogeneity, consistent with the expectations of the DSH.

## Methods

### General concepts

We regard “spatial heterogeneity” here as the manifestation of patchiness (discrete habitat patches) at a particular scale, which embodies the size of a patch and its relative isolation. Patch isolation can be defined in terms of Euclidean distance between patches or the relative permeability of the intervening matrix between patches (i.e., the effective isolation; Ricketts 2001). In the context of our model, a “landscape” consists of a single source patch (containing dispersing agents) and a single target patch (receptor of dispersing agents) separated in space by a particular (fixed) Euclidean distance or effective isolation. Landscapes can be defined at any spatial scale (as simply a spatially heterogeneous area; Turner 1989), and spatial scaling refers to a factorial change in both patch size and isolation. For analytical convenience, we make a number of simplifying assumptions with regards to dispersal. We do not explicitly consider various phases of dispersal, such as emigration and settlement (Royce 2007), and instead define “dispersal” as successful movement (number of individuals or propagules) from a source to a target habitat patch, which may incorporate “travel costs” (Travis et al. 2012) as a component of the effective isolation between patches. We do not differentiate the concept of “success” any further with regard to the relative abilities of dispersers in locating resources within the target patch, nor do we consider the fate of dispersing agents that fail to reach the target patch. Given these assumptions, dispersal results purely from an interaction between the scale of heterogeneity and the gap-crossing abilities of the species of interest. We thus view dispersal as a functional response to landscape structure (i.e., functional connectivity between source and target patches achieved through successful movement; Baguette and Van Dyck 2007).

### Dispersal kernels

Dispersal density (number per unit area) over a distance *r* (length) is the product of a source production term (number) and an “isotropic” density function, or dispersal kernel *f*(*r*) (per unit area). Isotropic dispersal (movement that is invariant with respect to direction) is a classic simplifying assumption made in theoretical ecology and ecological modeling, which serves as a first approximation from which departures resulting from the relaxation of assumptions (i.e., directed dispersal) can later be evaluated (Turchin 1998). We assume an exponential power distribution for the dispersal kernel, which has the advantage of a flexible shape (Clark et al. 1998; Clark et al. 1999; Fayard et al. 2009). The basic kernel is:

1 $$ f(r) = \frac{1}{N}{\exp}\left[ { - {{\left( {\frac{r}{\alpha }} \right)}^c}} \right] $$

where *α* is a distance parameter (meter), *c* is a dimensionless shape parameter, and *N* is a normalization constant:

2 $$ N = \int\limits_0^{\infty } {\oint_{{2\pi }} {{\exp}\left[ { - {{\left( {\frac{r}{\alpha }} \right)}^c}} \right]d\varphi dr = \frac{{2\pi {a^2}\varGamma \left( {2/c} \right)}}{c}} } $$

where *Γ* is the gamma function. The kernel can be concave at the source and leptokurtic (*c* ≤ 1), or convex and platykurtic (*c* > 1), or can incorporate other important and well-known density functions as special cases. Among the most common are the negative exponential (*c* = 1):

3 $$ f(r) = \frac{1}{{2\pi {\alpha^2}}}{\exp}\left( { - \frac{r}{\alpha }} \right) $$

the Gaussian kernel (*c* = 2):

4 $$ f(r) = \frac{1}{{\pi {\alpha^2}}}{\exp}\left[ { - {{\left( {\frac{r}{\alpha }} \right)}^2}} \right] $$

and the square root negative exponential (*c* = 1/2):

5 $$ f(r) = \frac{1}{{24\pi {\alpha^2}}}{\exp}\left( { - \sqrt {{\frac{r}{\alpha }}} } \right) $$

As a result of its flexibility, the exponential power distribution has been applied in a number of theoretical studies that address dispersal (Kot et al. 1996; Clark 1998; Clark et al. 1998, 1999; Austerlitz et al. 2004; Bianchi et al. 2009; Fayard et al. 2009; Estep et al. 2010; Crossman et al. 2011).

**Fig. 2:**
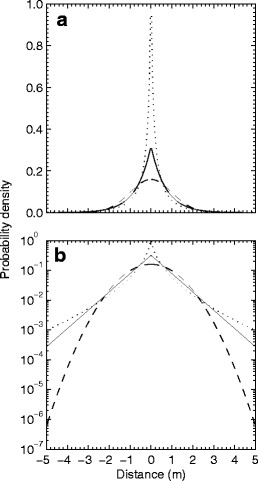
Exponential power distributions used for dispersal. Negative exponential (Eq. 3: *solid line*, *c* = 1), Gaussian (Eq. 4: *dashed line*, *c* = 2), and square root negative exponential (Eq. 5: *dotted line*, *c* = 1/2). Probability density is expressed on a linear *y*-axis in **a** and on a logarithmic axis in **b** to highlight differences in kernel shape. For demonstration purposes, the scale parameter is $\alpha = {{1} \left/ {{\sqrt {2} }} \right.}$for the negative exponential kernel, $\alpha = \sqrt {2}$for the Gaussian kernel, and $\alpha = {{1} \left/ {{\sqrt {{120}} }} \right.}$ for the square root negative exponential, resulting in a common variance of 1

The three kernels differ strongly in shape (Fig. 2). The Gaussian kernel appears as a bell shape if *f*(*r*) is plotted on a linear axis (Fig. 2a) and as a parabola when plotted on a logarithmic *y*-axis (Fig. 2b). The tails of the Gaussian kernel have the least probability mass, relative to the center of the distribution, of the three kernels. Differences in the thickness of kernel tails are best seen when probability density is plotted on logarithmic axes (Fig. 2b). The negative exponential kernel has less probability mass near the center than the Gaussian kernel but more in the tails. This kernel appears as a triangle on logarithmic axes. The square root negative exponential kernel has the least probability near the center among the three distributions and the most in the tails. Theoretical studies suggest that the shape of the dispersal kernel, in particular the fatness of the tail, has a major effect on an organism’s potential to spread (Kot et al. 1996; Clark et al. 1998). Both the negative exponential and the Gaussian kernels are said to be “thin-tailed,” meaning that the tails decline as fast as or faster than an exponential function (Madden et al. 2007). If the kernel is thin tailed, the population advances at a constant velocity (Mollison 1977). Ecologists interested in processes that operate at fine spatial scales, such as the foraging behavior of small organisms, often use thin-tailed kernels. In contrast, the decline of the tails of the square root negative exponential kernel is clearly less than exponential, and this type of kernel is accordingly called “fat-tailed.” This kernel has an advantage over some alternative fat-tailed kernels, such as power functions, because it does not have an infinite density at the source (Clark et al. 1999). Kernels with fatter tails (Eq. 5) lead to expansion in “leaps and bounds” ahead of the expanding wave, which means accelerating expansion (Mollison 1977; Shaw 1995; Kot et al. 1996). Ecologists interested in processes that operate at broad spatial scales, such as reforestation of habitat fragments and long-distance population spread commonly employ fat-tailed kernels.

### Influence of spatial scaling on dispersal among patches

**Fig. 3:**
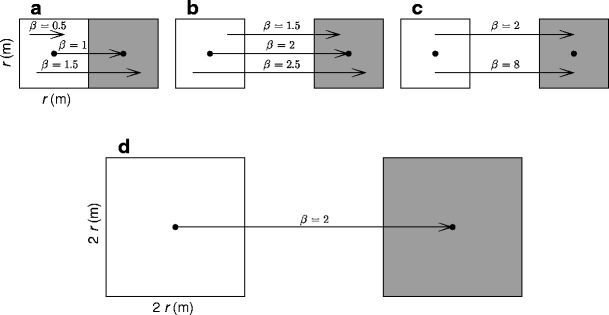
Spatial scaling of landscapes. Landscapes consist of a source patch (containing dispersing agents) and a target patch (receptor of dispersing agents), shown in white and gray, respectively. Source and target patches have dimensions *r* × *r* (square meter), giving a dispersal distance between the centroids of adjacent patches of *r* (meter). We use the parameter *β* (–) as a multiplicative factor of the dispersal distance *r*; the distance between source and target patches then becomes *β* *r*: **a** *β* can be used to provide dispersal to and from any point within or among patches that are adjacent, or **b** to and from any point between patches separated in space. Alternatively, **c** *β* can be used to approximate an intervening matrix between patches that is easy (*β* = 2) and difficult (*β* = 8) for the organism to traverse. Parameter *β* therefore alters the physical isolation of patches or their effective isolation owing to the resistance of the landscape to movement between patches, i.e., it is a patch isolation parameter. For a fixed value of *β*, **d** when we increase *r* we concomitantly scale patch size and distance between patches. We use four values of *β*: 1/2, 2, 8, and 32, to approximate varying degrees of patch isolation. For each value of *β*, we use a monotonically increasing series of *r* values to scale the spatial domain

We define a scale parameter *r* of dimension length (meter), and use this to facilitate the spatial scaling of source and target patches as well as the distance between them. We assume square source and target patches with dimensions *r* × *r* (square meter), giving a centroid to centroid gap distance between adjacent patches of *r*. Thus, as we increase *r*, we concomitantly scale the source and target areas as well as the distance between them. In order to alter the degree of association between patch size and distance between patches, we introduce *β* (–) as a multiplicative factor of gap distance *r*. The distance between a source and target patch with dimensions *r* × *r* then becomes *β* *r*. Various values of *β* can be used to consider movement between any two points within the source and target patches, or as a measure of the “effective isolation” between patches due to the resistance of the intervening matrix to dispersal (Fig. 3). Including parameter *β* therefore allows us to view the intervening matrix between patches from both a “structural” and a “functional” perspective: the Euclidean interpatch distance that must be traversed, which is a function of habitat configuration; or the “effective isolation distance’ that must be traversed, which incorporates the ability of organisms to move through an intervening matrix of differing resistance or permeabilities to movement (functional connectivity; Wiens et al. 1993; Ricketts 2001). We treat these two interpretations of the effects of *β* as synonymous, as in both cases *β* alters distance of movement (the Euclidean distance or effective isolation) and the likelihood of dispersal, and we use four values of *β* (1/2, 2, 8, and 32) to approximate varying degrees of patch isolation (Fig. 3). For analytical convenience, we assume that the number of dispersal agents is proportional to source area according to a density parameter, *k* (number per square meter) and that dispersers are a random sample of the population. Again, this is a common simplifying assumption in theoretical ecology from which departures resulting from additional complexities (e.g., variation in habitat quality) can later be evaluated. Under these simplifying assumptions, the number of individuals, *y* (number), dispersing from a source patch and landing in a target patch of the same area is given by the product of the source term, *k r* ^2^ (a positive dispersal force that increases with *r*; Fig. 1), the dispersal probability, *f*(*α,β* *r*) (a negative dispersal force that decreases with *r*; Fig. 1), and the target area, *r* ^2^ (a positive dispersal force that increases with *r*; Fig. 1). Although errors are generated by the discretization of spatial processes, this is common practice in the construction of parsimonious and analytically tractable ecological models. For a monotonic series of *r* values and a fixed value of *β*, we obtain a distribution of *y* values that reveals the relationship between the scale of spatial heterogeneity (patch size [*f*(*r*)], patch isolation [*f*(β *r*)]), and dispersal [*f*(α,β *r*)] (hereafter, scaling distribution of dispersal). Any dispersal model could be used, but for the density functions given previously (Eqs. 3–5), the resultant formulae are:

6 $$ y(r) = \frac{{k{r^4}}}{{2\pi {\alpha^2}}}{\exp}\left( { - \frac{{\beta r}}{\alpha }} \right) $$

7 $$ y(r) = \frac{{k{r^4}}}{{\pi {\alpha^2}}}{\exp}\left[ { - {{\left( {\frac{{\beta r}}{\alpha }} \right)}^2}} \right] $$

8 $$ y(r) = \frac{{k{r^4}}}{{24\pi {\alpha^2}}}{\exp}\left( { - \sqrt {{\frac{{\beta r}}{\alpha }}} } \right) $$

Empirically derived values for *k* and *α* from the literature were used to give a range of different dispersal modes for a variety of ecologically important organisms: (1) the mosquito *Culex erraticus*, an important vector for many pathogens including eastern equine encephalitis virus, which is considered to be the most dangerous endemic arbovirus in the USA (Jacob et al. 2010); (2) spores of the oomycete *Phytophthora infestans*, causative agent of potato late blight, widely regarded as one of the most costly constraints to attaining global food security (Evans and Waller 2010); and (3) red maple (*Acer rubrum*) seeds, one of the most abundant and widespread deciduous trees in eastern North America (Abrams 1998). Scaling distributions of dispersal were calculated for these three species using Eqs. 6–8, respectively, as the different dispersal kernels in these formulae have previously been tested for these species (Table 1).

**Table 1:** Parameters used to define dispersal processes

Dispersing agent	*c* (–)	*α* (m)	*k* (no. m^−2^)
*Culex erraticus* ^a^	1	1,603	22
*Phytophthora infestans* ^b^	2	2.78	2.28 · 10^6^
*Acer rubrum* ^c^	0.5	30.8	73,100

^a^ *c* and *α* from Estep et al. (2010), *k* from Ameen et al. (1994)

^b^ *c* and *α* from Skelsey et al. (2005), *k* from Skelsey et al. (2010)

^c^Clark (1998)

## Results

**Fig. 4:**
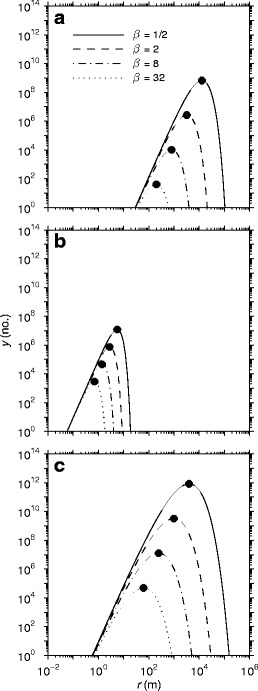
Scaling distributions of dispersal. Panels show the interaction between the scale of spatial heterogeneity (patch size [*f*(*r*)], patch isolation [*f*(*β* *r*)]) and the gap-crossing abilities of the organism on the number of redistributed agents among source and target patches, *y*, under different dispersal assumptions: **a** exponential dispersal (Eq. 6) of mosquitoes (*C. erraticus*) (Table 1); **b** Gaussian dispersal (Eq. 7) of *P. infestans* sporangia; and **c** square root negative exponential dispersal (Eq. 8) for red maple (*A. rubrum*) seeds. The set of *four curves* per panel show the influence of increasing patch isolation (Euclidean distance or effective isolation) on successful movement between source and target patches. *Solid data markers* show the global maximum values of Eqs. 6–8

The relationship between dispersal and scale of spatial heterogeneity was remarkably similar among organisms that exhibit vastly different modes of spread or transport (Eqs. 6–8 parameterized for real organisms; Fig. 4). Consistent with the conceptual model of the DSH (Fig. 1), the scaling distribution of dispersal was parabolic in form in each case, such that dispersal was maximized at an intermediate range of scales.

Increasing patch isolation (Euclidean distance or effective isolation between patches, *β*) served to decrease dispersal with a magnitude that increased with spatial scale (Fig. 4). At fine spatial scales, dispersal was relatively unaffected by patch isolation for these three organisms, as noted by the congruence among all four curves in this domain. At coarser spatial scales, however, there was a much larger effect of patch isolation on dispersal. Beyond the intermediate maxima, increasing isolation leads to decreased dispersal in spite of larger population sizes (=more dispersers) within these large patches, because the gap-crossing abilities of the organism are eventually exceeded by the increasing interpatch distances or resistance of the matrix to dispersal. Significantly, the effect of patch isolation on the scaling distribution of dispersal remains unchanged across a wide range of dispersal assumptions and scales (Fig. 4a–c). In all cases, the scaling distribution of dispersal is a parabolic function that attains a maximum value at an intermediate range of scales.

Finding the precise spatial scale of maximum dispersal for Eqs. 6–8 was a simple matter of finding the global maximum for those functions. Some basic calculus yields: 4*α*/*β*, $\alpha \sqrt {{{{2} \left/ {\beta } \right.}}}$, and 64*α*/*β* for Eqs. 6–8, respectively (Fig. 4). That these global maxima are a function of the dispersal distance and patch isolation parameters [*f*(*α*,*β*)] confirms that dispersal is maximized when the scale of spatial heterogeneity is neither too fine nor too coarse relative to the gap-crossing abilities of the organism.

## Discussion

That dispersal should be maximized at intermediate scales relative to the organism (DSH) is a novel hypothesis that formalizes the relationship between the spatial scale of patchiness and the gap-crossing abilities of species in spatially heterogeneous landscapes. That this scaling relationship holds across a diverse range of dispersal modes and landscape structures (landscapes with different gap properties) suggests that, regardless of the absolute length scales at which dispersal occurs, the relationship is a general and reasonably robust one.

It should be noted, however, that the scaling transition was dealt with in a linear way in this analysis, in that the same isotropic dispersal process was applied across a range of scales (patch sizes and distances between patches). Such a linear scaling relationship may not be applicable in all contexts. For example, animal movement may not exhibit a similar response to spatial pattern across all scales: foraging movements between small resource patches may differ from long-distance movement between different habitat patches or ecosystems. It could be argued that if an organism can travel between intermediate-sized patches with intermediate gap distances, it can also reach smaller patches separated by shorter gap distances, meaning that dispersal would start to become limiting at coarse scales but may not be reduced at finer scales. Alternatively, resources at finer spatial scales may fall below the perceptual range of the disperser, and therefore may not be detected (Wiens 1989; Kotliar and Wiens 1990; Lima and Zollner 1996). Thus, the fact that a linear scaling process led to a parabolic ecological response raises the question of whether such a non-linear scaling relationship for dispersal in patchy landscapes might lead to the evolution of optimal domains of movement behavior, where individuals maximize their success by operating within some intermediate range of scales bounded by patch size, gap properties and the extent of their perceptual resolutions? Although in this study we did not address the potential effects of animal behavior (or other forms of non-random dispersal) on predictions of the DHS, nor the potential effects of other aspects of spatial heterogeneity, such as clumping of habitat or variation in habitat quality, we have explored these complexities within a different modeling framework and can affirm that we obtain results that are qualitatively similar to those of the analytical model presented here (Skelsey et al., in review).

The notion of antagonistic forces being responsible for some maximum response level is generic to many different biological and physical systems. For example, the Janzen–Connell Hypothesis is a widely accepted explanation for the maintenance of tree species biodiversity in tropical tree communities (Janzen 1970; Connell 1971). It predicts that seed deposition decreases with distance from a parent tree, but those seeds that are deposited farthest from the parent have a competitive advantage as they are more distant from seed predators that are found more commonly around the parent. This leads to a maximum response in seedling recruitment at an intermediate distance from the parent tree. Obviously, what qualifies as an “intermediate distance” will depend on the tree species and the foraging range of its seed predators. The DSH predicts a similar response but without the need to invoke any form of interspecific interaction. Furthermore, just as the DSH predicts that dispersal is maximized when spatial heterogeneity is neither too fine nor too coarse relative to the gap-crossing abilities of an organism, in a similar vein, the intermediate disturbance hypothesis (IDH; Grime 1973; Connell 1978) predicts that species diversity is likely to be enhanced when ecological disturbance is neither too rare nor too frequent. This is because intermediate levels of disturbance allow both competitive K-selected and opportunistic r-selected species to coexist. What qualifies as an “intermediate level” of disturbance will depend on the type of disturbance and the relative sensitivity of different types of species to that disturbance within the system in question. Although the IDH is a simplification of the complex interactions that occur between species and their environment, it continues to be a useful framework for understanding the influence of disturbance on species diversity within communities (e.g., Roxburgh et al. 2004). We view the DSH as a similarly general framework for understanding the relationship between scale and dispersal, and what constitutes an “intermediate scale” is expected to be both species and landscape dependent.

The finding of intermediate-scale optima in dispersal in patchy landscapes has important implications, as many species in nature are distributed and operate across a wide range of spatial scales. It is therefore imperative that we recognize when and where dispersal is likely to have a pervasive influence—and when it does not. Understanding that intermediate scales have the potential to support the greatest connectivity among sites or populations is a step toward identifying the scales at which management can have greatest impact. As information regarding the dispersal characteristics of an organism or process becomes available, we can use the DSH to predict the scale at which connectivity is maximized or minimized (e.g., Eqs. 6–8). Such knowledge could prove to be invaluable in a wide variety of fields where management of dispersal or connectivity may be required: from disruption of the spread of invasive species and pathogens, to optimization of conservation reserve networks, to minimization of the environmental impacts of a host of anthropogenic activities, such as habitat loss and fragmentation caused by agriculture and industry.
